# TRPM8 mechanism of autonomic nerve response to cold in respiratory airway

**DOI:** 10.1186/1744-8069-4-22

**Published:** 2008-06-05

**Authors:** Hong Xing, Jennifer X Ling, Meng Chen, Richard D Johnson, Makoto Tominaga, Cong-Yi Wang, Jianguo Gu

**Affiliations:** 1 Department of Oral & Maxillofacial Surgery and Diagnostic Sciences, McKnight Brain Institute and College of Dentistry, University of Florida, Gainesville, Florida 32610, USA; 2 Department of Physiological Sciences, McKnight Brain Institute and College of Veterinary Medicine, University of Florida, Gainesville, Florida 32610, USA; 3 Section of Cell Signaling, Okazaki Institute for Integrative Bioscience, National Institutes of Natural Sciences, Higashiyama 5-1, Myodaiji, Okazaki, Aichi 444-8787, Japan; 4 Center for Biotechnology and Genomic Medicine, Medical College of Georgia, 1120 15th Street, CA4098, Augusta, GA 30912, USA

## Abstract

Breathing cold air without proper temperature exchange can induce strong respiratory autonomic responses including cough, airway constriction and mucosal secretion, and can exacerbate existing asthma conditions and even directly trigger an asthma attack. Vagal afferent fiber is thought to be involved in the cold-induced respiratory responses through autonomic nerve reflex. However, molecular mechanisms by which vagal afferent fibers are excited by cold remain unknown. Using retrograde labeling, immunostaining, calcium imaging, and electrophysiological recordings, here we show that a subpopulation of airway vagal afferent nerves express TRPM8 receptors and that activation of TRPM8 receptors by cold excites these airway autonomic nerves. Thus activation of TRPM8 receptors may provoke autonomic nerve reflex to increase airway resistance. This putative autonomic response may be associated with cold-induced exacerbation of asthma and other pulmonary disorders, making TRPM8 receptors a possible target for prevention of cold-associated respiratory disorders.

## Background

Normally, a breath of cold air is warmed up to near body temperature through heat exchange in the upper airway, mainly the nose, before the air enters the bronchopulmonary system. Temperature exchange, however, is compromised under conditions including flu, allergy, and other respiratory diseases. Exercise in cold weather can also result in the rapid inhalation of cold air into the trachea and bronchi, and the air temperature there can drop as low as about 20°C due to an insufficient temperature exchange [1,2]. Respiratory responses to cold air are reflexive, including cough, airway constriction and mucosal secretion. These responses may have some protective roles for bronchopulmonary tissues when exposed to potentially hazardous cold environment. However, the responses can be harmful in people having certain respiratory diseases. For example, cold is a major environmental factor that exacerbates existing asthma conditions and directly triggers asthma [3]. Inhalation of cold air is a direct cause of airway constriction to trigger exercise asthma in athletes performing winter sports [4,5]. Clinically, the "cold air challenge test", a test of bronchopulmonary reactivity and airway resistance, has been used for asthma diagnosis for over 20 years [6,7] because many asthma patients show bronchopulmonary hyper-reactivity and increased airway resistance to cold air challenge.

Respiratory responses to cold may be through a neural reflex mechanism [8,9]. The main afferent nerves that innervate the bronchopulmonary system are derived from the vagus nerve. Factors that stimulate these nerves trigger an autonomic reflex to cause airway constriction and mucosal secretion [10,11]. If respiratory responses to cold are indeed mediated by bronchopulmonary vagal afferents, what is the molecular mechanism by which cold initiates the autonomic responses?

Recently, studies have identified a molecular mechanism for sensing cold by the somatic sensory nerve endings of the skin [12-19]. It has been demonstrated that cool temperature opens a new type of ion channels (receptors) on the membranes of a subpopulation of somatic sensory nerves, which causes sensory nerve excitation [20,21]. The ion channels were cloned from somatic sensory neurons of rats [12], mice, and humans [13], and were named transient receptor potential channel M8 (TRPM8) [12,13]; since it belongs to the transient receptor potential (TRP) super-family. When expressed on heterologous cell systems, cooling temperatures below 24–28°C start to evoke depolarizing currents. TRPM8-mediated currents increase with decreasing temperatures and reach maximum currents near 10°C. TRPM8 can also be activated by menthol, the active ingredient of peppermint, and by other cooling compounds [12]. Electrophysiological studies have indicated that TRPM8 is highly permeable to Ca^2+ ^[12,13,21], and activation of TRPM8 results in a large increase of intracellular Ca^2+ ^levels [12,13,20-22] through both Ca^2+ ^entry from extracellular sites and Ca^2+ ^release from intracellular Ca^2+ ^stores [22].

Vagal afferent nerves and somatic sensory nerves are two different nervous systems. Functionally, somatic sensory afferent fibers sense stimuli to produce conscious sensations. On the other hand, vagal afferent nerves belong to autonomic nervous system and are not involved in any conscious sensation. Stimulation of vagal afferent nerves only produces autonomic reflex. However, several sensory molecules that are found in somatic sensory neurons are also found in vagal afferent nerves. For example, VR1 receptor (vallinoid receptor-1) is found in nociceptive somatic sensory fibers and serves as a sensor for noxious heat [23]. This receptor is also expressed on some vagal afferent nerves and activation of this receptor by capsaicin, an active ingredient of hot chili pepper can produce cough reflex and neurogenic inflammation in the bronchopulmonary system [24]. In the present study, we have tested the hypothesis that cold excites bronchopulmonary vagal afferent nerves through the activation of TRPM8 receptors.

## Methods

### Retrograde labeling and preparation of vagal ganglion neurons

Adult Sprague-Dawley rats (200 to 300 g, n = 48) were used according to the Institutional Animal Care and Use Committee guideline of the University of Florida. Retrograde labeling of the vagal ganglion (**VG**) neurons that innervate low airway tissues was performed based on a method described previously [25]. In brief, rats were continuously anesthetized with isoflurane using an anaesthetizing machine. A small amount of 1,1'-dioctadecyl-3,3,3',3'- tetramethylindocarbocyanine perchlorate (DiI, 20 μl, 0.25% in DMSO) was gradually instilled into the caudal region of rat trachea using a 50 μl Hamilton syringe. The animals were positioned supine during dye instillation and kept the same position for 30 min before recovery from anesthesia. Seven days after dye instillation, both left and right vagal ganglions (nodose ganglions) were harvested from the animals.

The acutely dissociated neurons were prepared in a manner described in our previous studies. In brief, the ganglions were incubated for 45 min at 37°C in S-MEM medium (Gibco, Grand Island, NY) with 0.2% collagenase and 1% dispase (Sigma) and then triturated to dissociate neurons. The VG neurons were then plated on glass coverslips previously coated with poly-D-lysine (PDL), maintained in normal bath solution (see below) at an ambient temperature of 26°C. Cells were used within 4 hours after plating. In a different set of experiments, acutely dissociated dorsal root ganglion (DRG) neurons were used and DRG cell preparation was performed in the same was as VG neurons [26]. DiI-labeled VG neurons were identified under the fluorescence microscope (excitation, 550 nm; emission, 650 nm).

### Ca^2+ ^Imaging

VG neurons were incubated with 2 μM Fluo3-AM for 30 min at 37°C to load Ca^2+ ^indicator. Fluo3-AM was prepared in 20% pluronic acid (Molecular Probes, Eugene, OR). The cells were then perfused with normal bath solution flowing at 1 ml/min in a 0.5 ml chamber. The normal bath solution contained (in mM): 150 NaCl, 5 KCl, 2 MgCl_2_, 2 CaCl_2_, 10 glucose, 10 HEPES, pH 7.2, osmolarity adjusted to 320 mOsm with sucrose. Fluo-3 fluorescence in the cells was detected using a peltier-cooled charge-coupled device (CCD) camera (PentaMAX-III System, Roper Scientific, Trenton, NJ) under a fluorescence microscope (10X objective). Excitation was at 450 nm and emission at 550 nm, achieved by a fluorescence filter sets. Images were taken at one frame per second, and digitized using MetaFluor software (Universal Imaging Corporation). Unless otherwise indicated, all experiments were carried out at an ambient temperature of 26°C. Effects of cold on intracellular Ca^2+ ^levels were tested by application of a cold bath solution, which yield temperature drop from 26°C to 19°C within 1 min in the recording chamber. Effects of menthol were tested by application of 100 μM menthol for 20 s. Cold and menthol solutions were delivered through a glass tube; the tube had internal diameter of 0.5 mm and was positioned 0.5 mm away from the recorded cells.

### Patch-clamp recordings

Whole-cell recordings were performed on VG neurons and DRG neurons. For most voltage-clamp recordings, electrode internal solution contained (in mM): 110 Cs_2_SO_4_, 2 MgCl_2_, 0.5 CaCl_2_, 5 TEA-Cl, 5 EGTA, 5 HEPES, pH 7.3. When both voltage-clamp and current-clamp recordings were applied to the same cells, electrode intracellular solution contained (in mM) 135 K-gluconate, 2 MgCl_2_, 0.5 CaCl_2_, 5 EGTA, 10 HEPES, 2 Na_2_ATP, 0.5 NaGTP, pH 7.3. Recording electrode resistance was ~5 MΩ. Unless otherwise indicated, voltage-clamp recordings were performed on cells held at -70 mV. Signals were amplified with MultiClamp 700A (Axon Instruments, Union City, CA), filtered at 2 kHz and sampled at 5 kHz. Cold bath solution and menthol were applied to neurons in the same manner as that in Ca^2+ ^-imaging experiments. A voltage ramp from -90 mV to 70 mV was used to obtain I-V relationship of menthol-evoked currents. The voltage ramp was applied during the steady states of menthol-evoked currents. I-V relationship was constructed after a subtraction of a control ramp test. In the voltage-ramp test, lidocaine (1 mM) was used to block both TTX-sensitive and TTX-resistant Na^+ ^channels and Ca^2+ ^channels [27].

### Immunostaining

Adult Sprague-Dawley rats (200–250 g, n = 6) were perfusion fixed and the inferior parts of vagal ganglions (nodose ganglions) were dissected out. The ganglions were cut with a cryostat into transverse sections at thickness of 16 μm. Sections were incubated in a rabbit anti-TRPM8 antibody (1:300) overnight at 4°C and then 3 hrs with a secondary antibody (goat-anti-rabbit, conjugated with Alexa 594). The TRPM8 antibody was generated in rabbits using a sequence of N-terminus, EGARLSMRSRRNG, of rat TRPM8 receptors. Double immunostaining of TRPM8 and P2X_3 _receptors were performed by first incubating ganglion sections with a guinea pig anti-P2X3 antibody (1:3000) overnight at 4°C, followed by 3 hrs of incubation with a secondary antibody (goat-anti-guinea, conjugated with Alexa 488). Immunostaining for TRPM8 receptors were subsequently performed as described above. Images were acquired using a fluorescence microscope. To determine immunoreactive positive neurons in each sample, a threshold is set at 2.5 times of averaged cytoplasmic density level. All neurons sectioned through their nucleus for which mean optical density exceeded the threshold were counted as positive.

### Data analysis

For Ca^2+^-imaging experiments, relative fluorescence intensity ΔF/F_0 _was used and a ΔF/F_0 _value of > 0.1 was considered to have response. All the data were represented as mean ± SEM. Paired-t tests were used for statistical comparison, and significance was considered at the p < 0.05.

## Results

**Figure 1:**
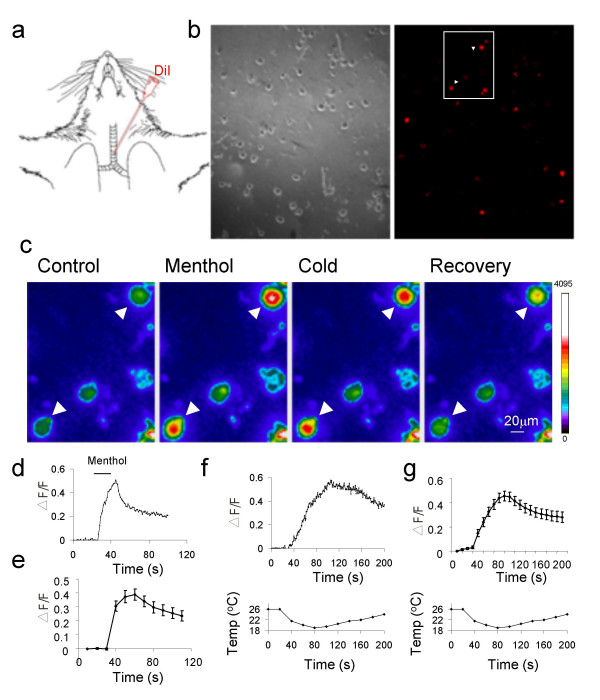
**Responses to menthol and cold in vagal ganglion neurons that innervate respiratory airways**. **a**, Diagram illustrates the instillation of DiI into the lower segment of rat trachea to retrograde label airway VG neurons. **b**, The micrographs show a field of acutely dissociated VG neurons under bright light (left) and fluorescence light (right). DiI-labeled VG neurons show strong fluorescence intensity. **c**, An example shows menthol- and cold-induced increases of [Ca^2+^]_in _in DiI-labeled VG neurons. The image was selected from the boxed region in (**b**) where two DiI-labeled VG neurons were included. Images were taken before (control), following a 20-s application of 100 μM menthol, following a 7°C temperature drop, and recovery in normal bath solution. **d&e**, Time course of menthol induced-responses in a DiI-labeled neuron (**d**) and pooled results (n = 28) (**e**). **f&g**, Time course of cold induced-responses in a DiI-labeled neuron (**f**) and pooled results (n = 27) (**g**). The fluorescence intensity changes (ΔF/F_0_) were shown on the top panels and temperature ramps on the bottom panels.

Seven days after the instillation of DiI into the respiratory airway (Figure 1a), the inferior parts of vagal ganglions (VG) were harvested from these animals and then dissociated for functional studies. Of 1638 acutely dissociated VG neurons, 170 of them showed a high intensity of DiI labeling (Figure 1b). We studied effects of the TRPM8 receptor ligand menthol [12,13] on the airway VG neurons using a Ca^2+ ^imaging technique. As shown in Figure 1c–e, application of menthol (100 μM, 20 s) resulted in increases of intracellular Ca^2+ ^in 7% of VG neurons (116/1638 cells), including both DiI-labeled and non-labeled neurons. Of the 170 DiI-labeled cells, 28 of them (17%) showed response to menthol (Figure 1c).

We determined whether cold stimulation might produce a response similar to menthol on VG neurons. Since there was little activation of TRPM8 receptors at temperatures above 24°C when cells were near resting membrane potential [28], a basal temperature of 26°C was used in our experiments. As shown in Figure 1c, f &1g, a 7°C of temperature drop, from 26°C to 19°C, resulted in increases of intracellular Ca^2+ ^in 6.5% of VG neurons (106/1638 cells), including both DiI-labeled and non-labeled neurons. All these cold-responsive cells were menthol-responsive neurons. Among the 170 DiI-labeled cells, 27 of them (16%) were both menthol- and cold-responsive neurons. These results indicate that a subpopulation of VG neurons innervating respiratory airways responds to both cold and the TRPM8 receptor agonist menthol.

**Figure 2:**
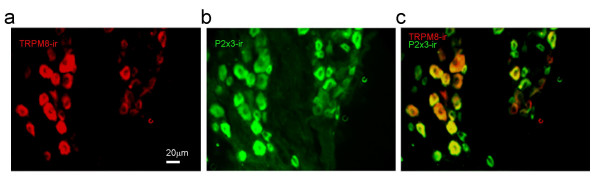
**TRPM8-immunoreactivity in VG neurons**. **a**, Image shows TRPM8 immunoreactivity (TRPM8-ir) in a portion of an inferior part of a VG section. TRPM8-ir neurons were not evenly distributed throughout the sections. **B**, Image shows P2X3 immunoreactivity (P2X3-ir) in the same VG section as in (**a**). **c**, An overlay image made with (**a**) and (**b**) shows co-localization of TRPM8-ir in many P2X3-ir positive neurons.

**Figure 3:**
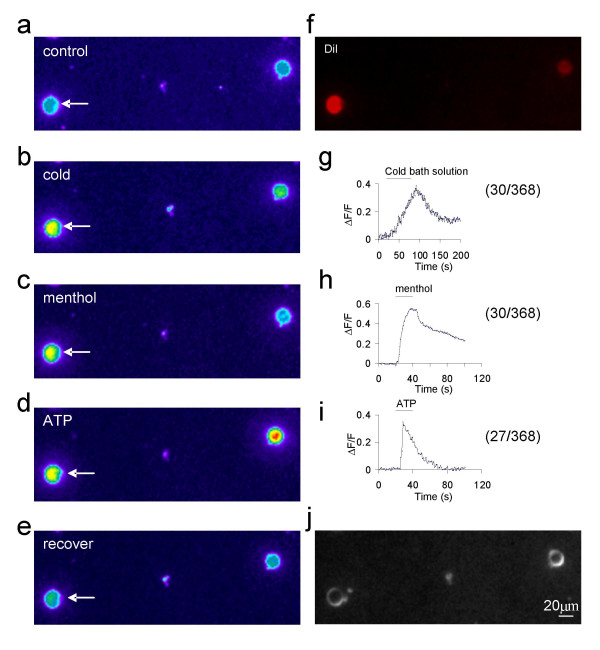
**ATP-sensitivity of cold- and menthol-responsive VG neurons**. **a-e**, Fluorescence intensity in a retrograde labeled VG neurons (arrow indicated) in control (a), following a temperature drop to 19°C (b), the application of 100 μM menthol (c), the application of 100 μM ATP (d), and recovery (e). **f**, The micrograph shows the DiI retrograde-labeled VG neurons. **g-i**, Fluorescence intensity changes over time in the cell shown in b, c, and d. **j**, Bright filed.

We next determined whether TRPM8 proteins were expressed on VG neurons by immunostaining sections of ganglions using a TRPM8 antibody. TRPM8 immunoreactivity was found to be present in 7.2% of VG neurons (75/1041 cells, Figure 2a). The cell sizes were 24 ± 0.7 μm, ranged from 12 to 36 μm. Many vagal afferent nerves innervating respiratory airways express P2X3 receptors and these nerves were suggested to be involved in vagal nerve reflex [29]. We determined whether TRPM8 receptors may be located on P2X3-expressing vagal neurons using double immunostaining with both the TRPM8 antibody and a P2X3 receptor antibody. About 40% of VG neurons (415/1041 cells, Figure 2b) were found to be P2X3-ir positive. The TRPM8-ir and P2X3-ir double positive neurons accounted for majority (68%) of TRPM8-ir positive neurons (Figure 2c). Furthermore, when menthol, cold, and ATP responses were tested using Ca^2+ ^imaging technique, we found that most menthol-responsive VG neurons were ATP-sensitive (Figure 3). Of 368 cells tested for cold, menthol and ATP, 30 of them were menthol- and cold-responsive cells. Of these 30 menthol- and cold-responsive cells, 27 of them were sensitive to ATP. These results indicate that TRMP8 receptors are expressed predominantly on a subpopulation of VG neurons that are ATP-sensitive.

**Figure 4:**
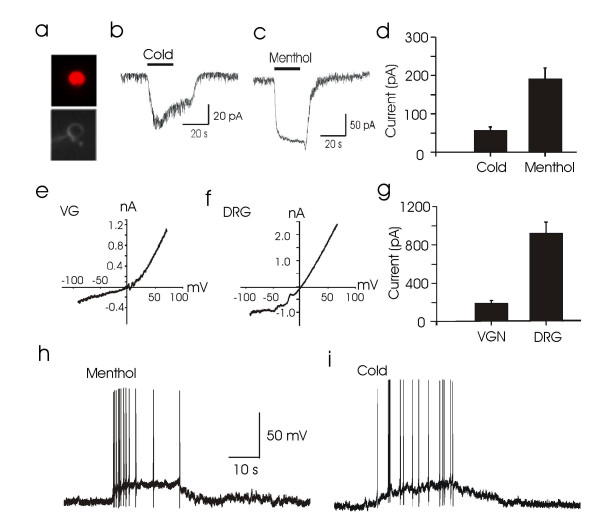
**TRPM8 receptor-mediated excitation of airway VG neurons**. **a**. Micrographs show a DiI-labeled VG neuron (top) and subsequent patched-clamp recording (bottom). **b**. A sample trace shows a whole-cell inward current evoked from the neuron by a 5°C of temperature drop within 20 s. **c**. Inward current evoked by 100 μM menthol in the same neuron as in (**b**). **d**. Pooled results from experiments shown in **b&c**. Holding potential was -70 mV in **b-d**. **e**. I-V relationship of menthol-evoked currents from a VG neuron. **f**. I-V relationship of menthol-evoked currents from an acutely dissociated DRG neuron. **g**. a comparison of menthol-evoked currents in VG neurons (n = 7) and DRG neurons (n = 21). Cells were held at -70 mV. **h&i**. Action potentials elicited by 100 μM menthol (**h**) and cold (**i**) in the same VG neuron. All recordings were made from menthol-sensitive neurons pre-identified using Ca^2+^-imaging technique. Menthol was applied at 100 μM for 20 s. Cold stimulation was achieved by a 20-s application of cold bath solution which yielded a 5°C temperature drop from 26 to 21°C.

To confirm that the responses of VG neurons to cold and menthol were indeed mediated by TRPM8 receptors, we characterized electrophysiological properties of cold- and menthol-induced responses. Figure 4a shows a DiI labeled VG neuron that was included in patch-clamp recording. All neurons used for the electrophysiological study were pre-identified to be menthol-responsive VG neurons using the Ca^2+ ^imaging technique. Under voltage-clamp configuration with cells held at -70 mV, a temperature drop from 26°C to 21°C within 20 sec evoked inward currents (Fig. 4b &4d, 57 ± 9 pA, n = 11). Menthol (100 μM, 20 s) was tested subsequently in 7 cells and all of them showed inward currents (Figure 4c &4d, 191 ± 29 pA, n = 7). The relationship between menthol-evoked currents and holding potentials showed strong outward rectification with a reversal potential near 0 mV (-2 ± 0.3 mV, n = 4, Figure 4e). These properties are consistent with the electrophysiological characteristics of the cloned TRPM8 that were expressed in heterologous expression system [12,13] as well as the characteristics of cold-sensing somatic sensory neurons acutely dissociated from rat dorsal root ganglions (DRGs) (Figure 4f). These results indicate that cold and menthol responses in respiratory airway VG neurons were mediated by TRPM8 receptors.

TRPM8 currents in VG neurons were found to be substantially smaller than those in DRG neurons (Figure 4g). Can TRPM8 activation be sufficient to excite VG neurons? To address this issue we made recordings on VG neurons under current-clamp configuration and tested the effect of menthol and cold. Menthol (100 μM, 20 s) depolarized VG neurons from the resting membrane potential of -60 mV (-60 ± 0.6 mV) to -38 mV (-38 ± 1.5 mV) and caused action potential firing (n = 4, Figure 4h). Similar to the response induced by menthol, a temperature drop from 26 to 21°C within 20 s depolarized VG neurons from the resting membrane potential of -61 mV (-61 ± 0.4 mV) to -36 mV (-36 ± 2.1 mV), which was followed by action potential firing (n = 4, Figure 4i). These results indicate that a 5°C of temperature drop to 21°C is sufficient to excite respiratory airway VG neurons.

## Discussion

In the present study, we have shown that a subpopulation of vagal afferent neurons innervating bronchopulmonary tissues expresses TRPM8 receptors and that cold can excite these autonomic afferent fibers through the activation of TRPM8 receptors. These findings provide a putative molecular mechanism by which cold induces autonomic responses in respiratory system. Thus, TRPM8 receptors have functions beyond encoding for consciousness of cold sensation in somatic sensory system.

Retragrade labeling with DiI used in this study allowed us to identify the vagal afferent neurons whose peripheral never endings innervated bronchopulmonary tissues. The cell bodies of these afferent fibers were used in the present work to study TRPM8 receptor expression and functions. Similar approaches have been used for studying expression and functions of other sensory receptors, e.g. P2X receptors, on vagal afferent neurons that innervate bronchopulmonary tissues [25]. Usually, a receptor that is used for detecting environmental stimulants is expressed on peripheral nerve endings as well as on their cell bodies. Sensory neuron cell bodies have been used as model systems to study sensory receptors because they have many advantages including the feasibility of using functional approaches such as calcium imaging technique and electrophysiological method. Nerve endings, on the other hand, are difficult to be studied directly using these functional approaches. Using calcium imaging approach, we have shown that 7% of neurons in the total neuron population responded to both menthol and cold. This is consistent with our immunostaining results. However, in the retrograde-labeled neurons, the cold- and menthol-responsive neurons were over 16%. This result suggests that bronchopulmonary system is a visceral tissue that is preferentially innervated by TRPM8-expression vagal afferent fibers. We have shown that most cold- and menthol-responsive neurons are also ATP-sensitive, suggesting that TRPM8-expressing neurons belong to polymodal sensory afferent neurons. The basal temperature for which cells were maintained was 26°C in the present study. Use of lower basal temperature is to minimize metabolic stress under *in vitro *experimental condition. In addition, TRPM8 receptors are not significantly activated at temperature above 24°C when cells were at resting membrane potentials [28], and thereby higher basal temperature is not necessary for the present study. We have shown that a few degree of temperature drop from 26°C results in significant increase of intracellular Ca^2+ ^and that all cold-responsive neurons are also sensitive to the TRPM8 agonist menthol. Patch-clamp recordings showed that menthol-evoked currents were outward rectified with a reversal potential near 0 mV. Thus, cold-induced response in these cells is mainly, if not absolutely, mediated by TRPM8 receptors.

The presence of TRPM8 receptors on neurons whose peripheral nerve endings innervate bronchopulmonary tissues predicts the expression of TRPM8 receptors on these nerve endings. Sensing cold temperature by these autonomic nerves and subsequent autonomic reflex may play a role in respiratory regulation responding to environmental temperature changes. In the earlier studies, TRPM8 receptors were reported to be only expressed on a subpopulation of dorsal root ganglion and trigeminal ganglion neurons in normal animals [12,13]. The expression of TRPM8 receptors on autonomic afferent nerves innervating bronchopulmonary system has not been reported previously. However, our finding is not completely unexpected because respiratory system opens to environments and sensing cooling temperatures can be physiologically important. In addition to bronchopulmonary vagal afferent fibers, recent studies have provided evidence suggesting that TRPM8 receptors are involved in bladder cooling reflex and micturition [30,31]. Thus, TRPM8 receptors may be widely expressed on afferent fibers innervating visceral organs and to be involved in reflexive responses.

Since only a small population of neurons expresses TRPM8 receptors, it raises a question as to whether TRPM8-expressing neurons randomly innervate airway trees, or whether there is a regional innervation of TRPM8-expressing neurons. Under physiological conditions, temperatures in the airway tree of the lung lobes are unlikely to drop below 35°C. However, temperatures in the upper airway such as laryngeal tracheal region may drop below 25°C. It is possible that TRPM8 receptors are mainly expressed on upper airway trees where these receptors serve as a sensor of cold temperatures to mediate reflexive responses.

An intriguing issue is whether TRPM8 receptors may be involved in cold-induced asthma and asthma exacerbation. This potential pathological function, if proven to be true, can be very significant clinically. Cold-induced asthma and asthma exacerbation is a well known phenomenon such that people susceptible to asthma are often advised to stay away from cold air and keep warm in winter season. In addition to cold, the factors responsible for the development of asthma also include genetic predisposition and other factors such as smoking and inflammation [32]. However, these factors often interact with each other to contribute to asthma etiology [32]. A number of previous studies in both animals and human have shown that cold increases airway resistance and the cold-induced responses were mediated by vagal afferent nerves [33-35]. In human, inhalation of cold air is known to be a direct cause of airway constriction in athletes performing winter sports, i.e. exercise asthma [4,36]. If TRPM8 is involved in cold-induced asthma and asthma exacerbation, it would be interesting to know whether TRPM8 receptor expression is up-regulated in those people who are susceptible to asthma such that they are more sensitive to cold than normal subjects. In addition to direct cold stimulation, inhalation of the vapors of menthol or peppermint oil can rapidly trigger airway constriction and asthma in some people [37,38]. However, low concentrations of menthol are often used for suppressing respiratory reactions [39]. Thus, TRPM8 receptors may have complicated respiratory functions under physiological and pathological conditions.

## Competing interests

The authors declare that they have no competing interests.

## Authors' contributions

HX carried out the calcium imaging and electrophysiological recordings. JXL performed immunochemical experiments. MC performed DiI injection and assisted electrophysiological recordings. RDJ guided the dissection of nodose ganglia. MT provide TRPM8 antibody and instructed immunochemical experiments. CYW participated in data analysis. JG designed the experiments, interpreted the data and wrote the paper.
